# Adipose stromal/stem cells assist fat transplantation reducing necrosis and increasing graft performance

**DOI:** 10.1007/s10495-013-0878-7

**Published:** 2013-07-05

**Authors:** Maria Serena Piccinno, Elena Veronesi, Pietro Loschi, Marco Pignatti, Alba Murgia, Giulia Grisendi, Ilaria Castelli, Daniela Bernabei, Olivia Candini, Pierfranco Conte, Paolo Paolucci, Edwin M. Horwitz, Giorgio De Santis, Lorenzo Iughetti, Massimo Dominici

**Affiliations:** 1 Division of Pediatric Oncology, Hematology and Marrow Transplantation, Department of Medical and Surgical Sciences for Children & Adults, University of Modena and Reggio Emilia, Modena Policlinic, Modena, 41100 Italy; 2 Division of Oncology, Department of Medical and Surgical Sciences for Children & Adults, University of Modena and Reggio Emilia, Modena Policlinic, Modena, 41100 Italy; 3 Unit of Plastic Surgery, Department of Medical and Surgical Sciences for Children & Adults, University of Modena and Reggio Emilia, Modena Policlinic, Modena, 41100 Italy; 4 Division of Hematology, Department of Medical and Surgical Sciences for Children & Adults, University of Modena and Reggio Emilia, Modena Policlinic, Via del Pozzo, 71, Modena, 41100 Italy; 5 Division of Oncology, Children’s Hospital of Philadelphia, Philadelphia, PA 19104 USA

**Keywords:** Adipose tissue, Autologous fat transfer, Necrosis, Mesenchymal stromal/stem cells, Lipopreservation

## Abstract

Autologous fat transfer (AFT) is a procedure for adipose tissue (AT) repair after trauma, burns, post-tumor resections and lipodystrophies still negatively impacted by the lack of graft persistence. The reasons behind this poor outcome are unclear and seem to involve damages in either harvested/transplanted mature adipocytes or on their mesenchymal progenitors, namely adipose stromal/stem cells (ASC), and due to post-transplant AT apoptosis and involution. A rabbit subcutaneous AT regeneration model was here developed to first evaluate graft quality at different times after implant focusing on related parameters, such as necrosis and vasculogenesis. Standard AFT was compared with a strategy where purified autologous ASC, combined with hyaluronic acid (HA), assisted AFT. Five million of autologous ex vivo isolated CD29+, CD90+, CD49e+ ASC, loaded into HA, enriched 1 ml of AT generating an early significant protective effect in reducing AFT necrosis and increasing vasculogenesis with a preservation of transplanted AT architecture. This beneficial impact of ASC assisted AFT was then confirmed at three months with a robust lipopreservation and no signs of cellular transformation. By a novel ASC assisted AFT approach we ensure a reduction in early cell death favoring an enduring graft performance possibly for a more stable benefit in patients.

**Electronic supplementary material:**

The online version of this article (doi:10.1007/s10495-013-0878-7) contains supplementary material, which is available to authorized users.

## Introduction

The autologous fat transplantation/transfer (AFT) has been performed since the 1890s and in the last 20 years the interest on this technique is enormously increased [1, 2]. In spite various harvesting and preparation techniques have been implemented to provide performing fat grafts, the outcome of AFT still needs optimization [3]. One of the greatest and unclear issues is related to graft reabsorption, arriving to 90 % and leading to either overcorrection or additional fat grafting negatively impacting the procedure [3, 4]. Recent studies suggest that, after lipoaspiration, more than 30 % of harvested mature adipocytes and up to 50 % of AT progenitors are lost due to both mechanical disruption and to localization of progenitor cells in perivascular regions of mid-size and large vessels that are generally spared during lipoaspiration [5, 6]. Beside the quality of the graft, even post-transplant events are currently under investigation, in particular regarding apoptosis and necrosis that have been frequently detected after AFT [7]. All together these findings may explain why transplanted AT does not stably persist leading to long-term atrophy.

In order to improve this outcome, Yoshimura group early developed a strategy where aspirated fat was supplemented by a heterogeneous pool of adipose derived cells defined as the stromal vascular fraction (SVF), consisting in freshly isolated cell mixture obtained through collagenase digestion [8]. This procedure has been introduced for breast augmentation in post-mastectomy breast reconstruction, as rescue for breast implant complications and for the treatment of severe lipoatrophies [9–11]. These studies underline that SVF may be a promising adjuvant population boosting the efficacy of conventional AFT. However, there seems to be a lack of standardization in the technique due to poorly controlled factors that may introduce biases, such as different devices to isolate SVF, sub-standardized adherence between SVF and AFT, their number and the same heterogeneous nature of SVF [6, 12].

Starting from this background, we sought to address a new strategy where more purified AT progenitors, namely mesenchymal adipose stromal/stem cells (ASC) [13, 14], could assist AFT investigating safety, efficacy and possible advantages in introducing a more defined population of AT progenitors coupled with fat graft. Thus, a subcutaneous fat regeneration approach was developed in rabbit to evaluate the graft survival in both a short and long term observation focusing on related parameters, such as apoptosis and vasculogenesis. By an autologous model we here begin to understand the early and long-term outcome of AFT, in parallel establishing a novel regenerative medicine approach able to counteract the early apoptotic events in transplanted tissue better supporting AT survival in vivo.

## Materials and methods

### Surgical procedures

**Fig. 1:**
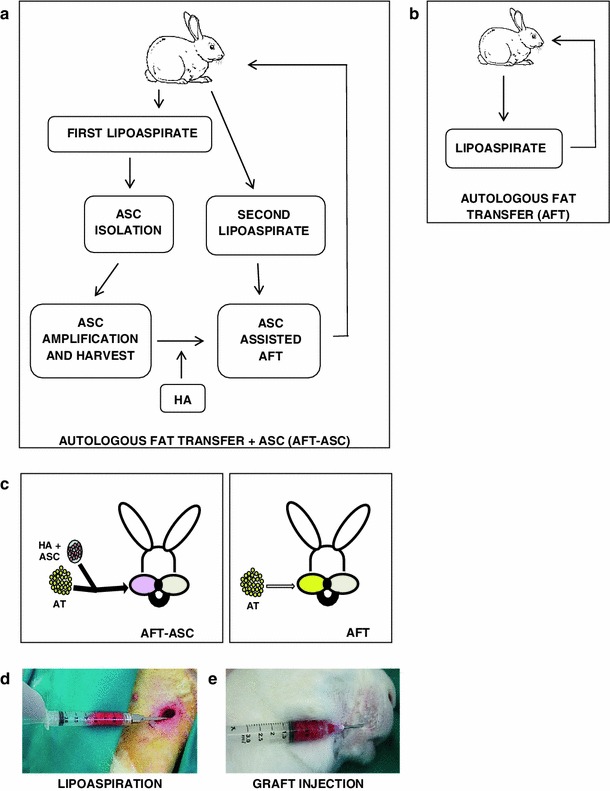
Experimental groups. **a**–**c** Animals were divided in two groups (*n* = 10/each), one received autologous fat transfer (AFT) supplemented by adipose mesenchymal stem cells (ASC) defined as AFT-ASC group (*panels a* and *left sided* **c**). Control group received autologous fat transfer only (AFT group; *panels b* and *right sided* **c**). The AFT-ASC group underwent to a first liposuction from the left inguinal fat pad to generate ASC. After 30 days the AFT-ASC groups underwent a second liposuction from the contralateral right inguinal fat pad and, after tissue decantation, 1 ml of compact lipoaspirate was combined with 200 μl of suspension containing 5 × 10^6^ ASC embedded into hyaluronic acid (HA). The obtained AFT-ASC composite was then injected in the right upper lip and equally distributed in subcutaneous position. AFT animals received standard autologous fat transplant into the right upper lip without ASC. **d** Lipoaspirate collection from inguinal fat pad. **e** Graft injection

Six months-old New Zealand female rabbits (Harlan Laboratories Srl, San Pietro al Natisone, Italy) weighing 4.5–5.0 kg (*n* = 20) were operated at the interdepartmental animal facility of the University of Modena and Reggio Emilia. The study protocol was approved by the local ethical animal Committee. Animals were divided in two groups (*n* = 10/each), a group (AFT-ASC) received autologous fat graft supplemented by ASC and hyaluronic acid (HA) while a second group received autologous fat graft only (AFT) as control. For the experimental outline see caption on Fig. 1a–c.

In the AFT-ASC group, the first lipoaspirates were performed from the left inguinal fat pad (Fig. 1d), as described [15]. Each rabbit was anesthetized with 3 % Sevorane (Abbott S.r.l., Latina, Italy), and intramuscular antibiotic (BAYTRIL, Bayer HealthCare LLC Animal Health Division, Pittsburgh, USA) was given as profilaxis. After preparatory procedures and skin sanitization, the inguinal fat pad was infiltrated with 3 ml of saline solution (0.9 % NaCl, S.A.L.F. S.p.A, Cenate Sotto, Italy) without injecting local anaesthetics or adrenaline. A small stab incision was performed and, according to Coleman technique [16], the blunt-tip cannula was inserted into inguinal fat pad and connected to a 3 ml luer lock syringe (BD, Frankling Lakes, USA). Incision was closed with 5-0 Ethilon (Johnson & Johnson, Auneau, France) suture and subsequently antibiotic ointment was applied. The procedure was lasting 15 min and each lipoaspirate (up to 2 ml) was then sent to the laboratory for ASC isolation. After 30 days from the first fat harvest, the same rabbits underwent a second operation for additional lipoaspirate collection from the contralateral inguinal fat pad. After AT decantation, 1 ml of lipoaspirate was gently mixed with a 200 μl buffer containing 5 × 10^6^ previously isolated ASC embedded in HA (Otihyal 1.6, OTI, Carsoli, Italy) carrier generating a combination of cells, AT and HA. This combination was then subcutaneously injected into upper-right lip as indicated in Fig. 1e using a 18 gauge needle (Chemil S.r.l., Padua, Italy). Site was then closed with 5-0 Ethilon suture (Johnson & Johnson) serving also as a marker for histological assessments, collectively transplant procedures last an average of 35 min. In the control group (AFT; Fig. 1b), only one single lipoaspirate was performed and 1 ml of decanted AT was reinjected into right upper lip without ASC. In both groups, contralateral left lip was preserved as internal matched control.

After transplantation, both AFT-ASC and AFT only groups were further sub-divided into two groups. One (*n* = 5) was sacrificed 7 days post-operatively for an early assessment, and the second after 3 months for a longer observation time. AT harvest was performed after shaving and, for transplanted animal only, the suture of the right lip was kept as marker for the injection site as described.

### Isolation and expansion of autologous rabbit ASC

Adipose samples taken for left inguinal fat pad, were washed in phosphate buffered saline PBS (PAA Laboratories GmbH, Pasching, Austria) minced and digested for 15 min in Dulbecco’s modified Eagle Medium (DMEM; Euroclone Spa, Milan, Italy) containing collagenase solution (Roche Diagnostics GmbH, Mannheim, Germany), 1 % of penicillin–streptomycin (P/S; 10^4^ UI/ml and 10 mg/ml, PAA) at 37 °C in 50 ml centrifuge tube by gentle agitation, as described [17]. The isolated cells were then centrifuged at 416 g for 10 min and enzyme activity was inhibited by adding DMEM plus 10 % heat-inactivated foetal bovine serum FBS (PAA). The resulting cell suspension was then centrifuged, filtered through a 100 μm cell strainer (BD Falcon, Durham, USA), counted by 0.4 % trypan blue exclusion (Biochrom AG, Berlin, Germany) and inoculated in culture flasks (Greiner Bio-One GmbH, Frickenhausen, Germany) at the density of 100,000 cells/cm^2^ with Quantum 333 (PAA) as culture medium. The cells were kept in incubators with a controlled atmosphere (5 % CO_2_, 37 °C) and the medium was replaced every 2–3 days, discarding non-adherent cells. Once 80–90 % confluence was reached, cells were detached with trypsin 0.05 %/EDTA 0.02 % (Euroclone), counted and seeded at 6,000 cells/cm^2^. Extended cultures were also protracted until reaching passage (P) 4. At each passage, cells were counted using 0.4 % trypan blue (Biochrom AG) exclusion, to evaluate viable cell expansion. When cells reach passage 4 we calculate doubling cells (DC) follow the formula: DC = Log_10_(P_i+1_/P_i_)/(log_2_10) where Pi means number of cells in each P in vitro [18].

### CFU-F assay

To assess the clonogenic potential of cultured MSC, the ASC fraction was additionally seeded for a fibroblastic colony-forming unit (CFU-F) assay. Collagen digested AT derived cells were seeded at a density of 100,000/cm^2^ and Quantum 333 (PAA) medium changed weekly, discarding nonadherent cells. MSC clonogenic precursors (CFU-F) were quantified after 10 days by Crystal Violet staining. Briefly, methanol solution with 0.4 % Crystal Violet (v/v) (Sigma-Aldrich, St. Louis, USA) was loaded into the flasks and staining was performed. Images were then collected by inverted microscope Axio Observer A1 with Plan-NeoFluar 10× objective and attached Axiocam MRC5 color camera (all Zeiss, Oberkochen, Germany). Clones of more than 50 cells were considered to be colonies. CFU-F assay allowed to calculate the population doubling (PD) of primary culture according the following: PD = Log_2_(E/I) where E was the number of cells at the end of primary culture and I was the number of seeded cells*CFU-F into SVF cells. All experiments were performed in triplicate for each animal.

### Fluorescence-activated cell sorting analyses (FACS)

Culture expanded ASC samples were detached from plastic support with trypsin/EDTA (Euroclone) counted and aliquoted in FACS analysis polypropylene tubes (0.5–1 × 10^6^/tube) (Falcon). ASC were subsequently incubated in the blocking buffer (100 μl each 0.5–1 × 10^6 ^cells) containing DMEM, 10 % FBS, 0.1 M Sodium Azide and human immunoglobulin G (Sigma) and incubated for 20 min on ice. After PBS wash (PAA), cells were stained in ice for 30 min with primary monoclonal antibodies (MoAb) in PBS with 0.1 % Bovine Serum Albumin (BSA, Sigma) and then with secondary antibody, where applicable. ASC (1 × 10^6^) were labelled with the following monoclonal antibodies (Table 1): FITC conjugated anti-rat CD90 (Biolegend, Milan, Italy), unconjugated anti-rat CD45 (Genetex, Milan, Italy), unconjugated anti-human CD29, CD49e, and CD105 (all from Serotec, Milan, Italy), APC conjugated anti-human CD10 and PE conjugated anti-human CD73 (BD) unconjugated anti-rabbit CD31 and CD146 (Abcam, Cambridge, UK). The appropriate isotype controls (BD Pharmigen and BD) and APC conjugated secondary antibody (BD) were introduced where applicable. The ASC were analysed by FACScalibur equipped with CellQuest software (BD) and 10,000 events were acquired.

**Table 1:** Antibodies for rabbit ASC characterization by FACS

Antigen	Host	Clone	Dilution	Manufacturer	Reported cross reactivity
CD29	Mouse	12G10	1/20	AbDserotec	No
CD49e	Mouse	JBS5	1/20	AbDserotec	No
CD90 FITC	Mouse	OX-7	1/30	Biolegend	Yes
CD10 APC	Mouse	HI10a	1/20	BD	No
CD31	Mouse	JC/70A	1/50	Abcam	Yes
CD45	Mouse	L12/201	1/20	GeneTex	Yes
CD73 PE	Mouse	AD2	1/20	BD	No
CD105	Mouse	SN6	1/20	Serotec	No
CD146	Mouse	P1H12	1/500	Abcam	Yes
IgGk1 anti mouse APC^a^	Goat	Polyclonal	5/100	BD	No

^a^Secondary ANTIBODY

### Differentiation assays

Cultured ASC were tested for their ability to differentiate into the main mesodermal lineages as previously described [17, 18]. Briefly, ASC were seeded in bone induction medium with DMEM (Euroclone) containing 10 % FBS (Hyclone, Logan, USA), 1 % P/S and glutamine (2 mM; Euroclone, Padmington, UK) supplemented with dexamethasone (10 nM), l-ascorbic acid-2-phosphate (0.1 mM), beta-glycerol phosphate (2 mM) (all Sigma) and bone morphogenic protein BMP2 (100 ng/ml; PeproTech, Rocky Hill, NJ, USA). After 2 weeks of induction, differentiated ASC and controls were stained with ddH2o 0.5 % Alizarin Red-S (v/v; Sigma). ASC were induced towards the adipogenic lineage using DMEM with addition of 1 % P/S, 10 % rabbit serum (Euroclone) and 5 % horse serum (Hyclone) supplemented with dexamethasone (1 mM), indomethacin (60 mM), rh-insulin (10 mM) and isobutylmethylxantine (0,5 mM; all from Sigma). ASC were maintained in differentiation media for 10 days and visualization of adipocyte differentiation was performed with Oil Red O (Sigma).

After induction differentiated cells and controls were visualized by microscopical observations (Axio Observer A1 with Axiocam MRC5 color camera and Axiovision 4.82 software; all Zeiss).

### Blood and sera sampling

Rabbit whole blood was collected from ear vein by a 23G needle syringe, placed into a serum clot activator tube (Greiner Bio-One, Caravaggio, Italy), and centrifuged at 2,175 g at room temperature for 15 min. Rabbit sera were collected and stored at −20 °C until autologous use.

### ASC assisted autologous fat transplantation (AFT-ASC)

To enrich AT with previously isolated ASC and HA the following procedure was adopted. Two hundred microlitre of carrier composed by 25 % HA hydrogel (HA; Otihyal 1.6, OTI, Carsoli, Italy), 25 % rabbit autologous serum and 50 % of saline solution (Laboratori Diaco biomedicali S.p.A., Trieste, Italy) were mixed with ASC. Otihyal 1.6 % is a pure unmodified HA sodium salt (molecular weight of 1 × 10^6^ Da) at 16 mg/ml of concentration. HA was prior incubated in 25 % of saline solution (Diaco) at 37 °C for about 18 h in a 1-ml tuberculin syringe (Artsana, Como, Italy). 5 × 10^6^ cells were suspended in 100 μl of saline solution additioned with rabbit autologous serum and then mixed into tuberculin syringe previously loaded with HA. Samples were then incubated at 37 °C for 10 min and combined with 1 ml of autologous concentrated lipoaspirate. Timely, after gentled mixing, the combination was injected into upper right lip.

### Histology

Histology was performed on both grafts and harvested tissues after transplantations. Triplicate samples of lipoaspirate, cell-gel and lipoaspirate-cell-gel were prepared for histological analyses to assess the interaction between cells, carrier and AT. Acid formalin (AF), composed by 10 % formalin diluted with 70 % ethanol and 5 % glacial acid (all Carlo Erba Reagenti SpA, Milan, Italy) was here introduced for its capacity to induce HA solidification [19]. After fixation, lipoaspirate, ASC-HA and lipoaspirate-ASC-HA were embedded in paraffin, 5-μm thickness sections were obtained and stained with Hematoxylin and Eosin (H&E; Carlo Erba) and Alcian Blue solution 1 % (w/v in 3 % acetic acid) pH 2.5 (Sigma).

Rabbit explants were instead fixed in 10 % neutral buffered formalin (NBF; Sigma), embedded in paraffin and 5-μm thickness sections were obtained and stained by H&E or Alcian Blue.

To detect vessels into transplanted tissues, immunohistochemistry using anti-CD31 antibody was performed accordingly a previously described protocol [20]. Five micrometer paraffin sections were dehydrated and stained with mouse-anti rabbit CD31 (1:20; Abcam) using a goat anti-mouse biotinylated secondary Ab (1:200; Vector Laboratories, Burlingeme, USA) and an avidin-biotin-horseradish peroxidase detection system (Vector). Retrieval were performed by proteinase K 2μg/ml (Promega Corporation, Madison, USA) for 5 min at room temperature and nonspecific binding were blocked using 10 % new calf serum and 10 % blocking reagent (Sigma). The primary antibody was applied overnight in a 0.1 % albumin bovine serum (BSA, Sigma) and 0.4 % triton X (Sigma). Following incubation with secondary Ab (Vector) and quenching solution, slides then were incubated with Vectastain ABC (Vector) as suggested by manufacturing instructions. Color development was performed by NovaRED (Vector) and slides were counterstained with Harris Hematoxylin (Bio Optica, Milan, Italy). Negative control specimens were stained with a mouse isotypic IgG primary Ab (Vector). Stained slides were then examined by Zeiss Axioskop (Zeiss). Photomicrographs were acquired by Axiocam IcC3 color camera and Axiovision 4.82 software visualization (Zeiss). Analyses were performed considering replicate specimens (*n* = 3) from each animal in both AFT-ASC treated and control groups using 100× magnification. Scoring was performed by counting CD31+ cells/100× high power field (*n* > 6/each specimen).

To assess the quality of AT after 3 months from the transplant, Sirius Red staining was additionally introduced, 5-μm paraffin sections were dehydrated and stained for 10 min with Carazzi Haematoxylin (Carlo Erba) and then Sirius Red (0.1 % in picric acid; Sigma) for 1 h of room temperature. After two washes in acidified water (0.5 % v/v in acetic acid, Carlo Erba), slides were mounted with coverslips and observed by microscopical examination.

Sirius Red selectively stains collagen fibres which are widely present in the connective subcutaneous region leaving un-stained both interstitial spaces and the intracytoplasmic non-fibrillar cell content, such the one of adipocytes. Thus, AT quantification was performed by reverse selection analysis of Sirius Red negative regions compared with the whole field by public ImageJ (http://rsb.info.nih.gov/ij/) software. The interstitial spaces and other un-target areas were not included in the analyses that were performed considering replicate specimens (*n* = 3/each animal) with 4 high-power fields (25×) from all animals in both AFT-ASC treated and control groups (Axio Observer A1 with attached Axiocam MRC5 color camera and Axiovision 4.82 software; all Zeiss).

### TUNEL assay

To assess graft viability post-transplantation, TUNEL assay was performed on rabbit specimens comparing grafted right tissues in both AFT and AFT-ASC groups with left untransplanted sites as control. Slides were stained by the DeadEnd Colorimetric TUNEL System (Promega) accordingly to the manufacturer’s instructions. Sections were counterstained with Harris Haematoxylin (BioOptica, Milan, Italy), observed by microscopical examination (Axio Observer A1) and images were analysed by software ImageJ in order to quantify the staining. The percentage of positive areas into the grafts was performed on at least 3 high power fields (25×) for each specimens (*n* = 3/each animal) and measured by the ImageJ software analysis.

### Statistical analyses

Data are expressed as mean values ± standard error of the mean (SEM). Statistical significance was determined by a two-tailed Student *t* test. A *p* value of < 0.05 was used for define the statistical significance.

## Results

### Inguinal lipoaspiration is a feasible and safe procedure in rabbit

All rabbits subjected to lipoaspirate from inguinal regions were healthy and small incisions healed in 7 days with no infections or complications including post-operatively bleeding, haematoma or seroma accumulation. From each procedure, in both experimental and control groups, up to 2 ml of lipoaspirate were harvested (Fig. 1d).

### Inguinal rabbit fat pad is a rich source of ASC

**Fig. 2:**
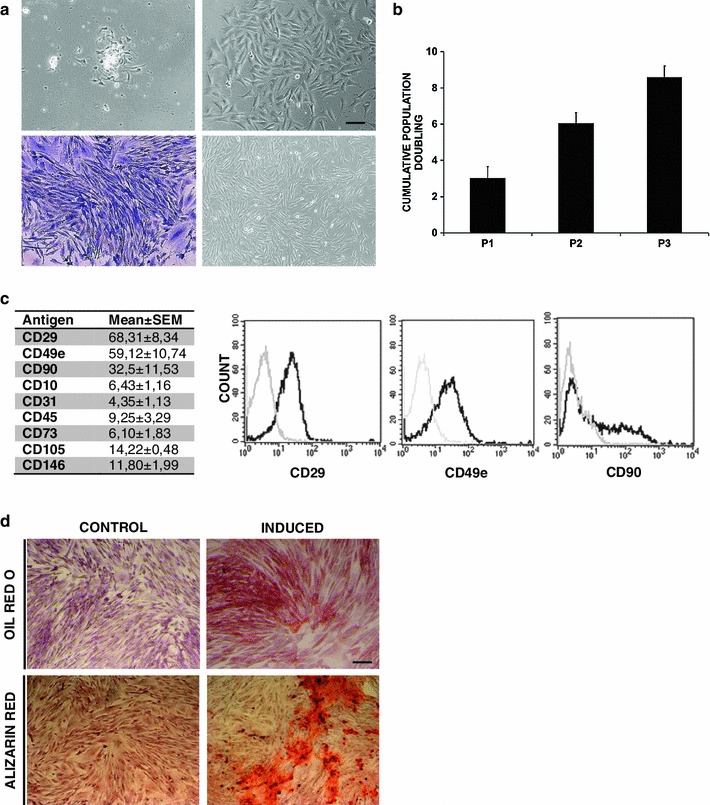
Rabbit fat pad originate performing ASC in vitro. **a** Photomicrographs of fibroblastoid elements adhering to plastic after adipose tissue digestion (*upper left panel*) subsequently generating colonies (*upper right panel*) visualized by Crystal Violet staining (*lower left panel*) and reaching confluence after three passages (*lower right panel* Ph1, *scale bar* 100 μm). **b** Rabbit ASC cumulative population doubling after passage 3(P3). **c** Mean values ± SEM of antigen expressed on rabbit ASC by FACS (*left table*); representative immunophenotypical characterizations of rabbit ASC with cells positive for CD29, CD49e and CD90 (isotype control in gray)**. d** Adipogenic differentiation after 10 days of induction visualized by Oil Red O staining and a corresponding non-induced control (*upper panels*; *scale bar* 100 μm). Representative osteogenic differentiation visualized by Alizarin Red staining after 14 days of induction and a corresponding non-induced control (*lower panels*)

AT specimens (*n* = 10), were processed for ASC isolation. An average of 152.74 ± 21.01 mg was processed, and after digestion, 5.56 ± 1.54 × 10^6^ cells were obtained and in vitro seeded. Forty-eight hours after seeding, fibroblastoid elements began to adhere to the plastic surface (Fig. 2a upper left panel) and after 6 days of culture early colonies were detectable (Fig. 2a upper right panel). After 10 days those clusters generated fibroblastoid-colony forming unit (CFU-F; Fig. 2a lower left panel), representative of a clonogenic potential of seeded cells as described for human MSC [18]. This was further confirmed quantifying those progenitors by crystal violet stain providing a mean value of 459 ± 212 CFU-F/100 mg AT. Cultures were then protracted and cells were further amplified (Fig. 2a, lower right panel). At the first passage the population doubling for the primary culture was 10.45 ± 0.68 calculated considering the CFU-F number. Fibroblastoid plastic adherent cells ended passages (P)3 with 8.49 ± 0.36 cumulative population doublings, indicating a relevant proliferation potential of isolated cells. Fibroblastoid shape and adhesion to plastic are recognized criteria to characterize a stem cell population, but others cell types like monocytes and endothelial cells maybe contaminants ASC preparations [21]. Therefore, a surface phenotypical characterization was attempted to define the nature of isolated cells. While for human or mouse MSC this has been fairly established, rabbit MSC are still poorly defined under the phenotypical point of view. After 4 passages, FACS analyses were performed introducing a panel of 9 distinct MoAb: CD90, CD29, CD49e, CD105, CD73, CD10, CD31, CD146 and CD45. As seen on Fig. 2c, rabbit ASC were positive for CD29 (68.31 ± 16.69) and CD49e (59.12 ± 21.47) while CD90 showed and intermediate expression (32.50 ± 11.53) suggesting new a panel for rabbit ASC characterization.

Having defined ASC phenotype, we then sought to assess rabbit ASC multilineage potentials. Thus, P4 ASC were incubated with specific culture media to induce both osteogenic and adipogenic differentiations in comparison with un-induced controls. After 10 days of adipogenic induction, we were able to observe round lipid droplets inside the cells and Oil Red O staining confirmed the adipose commitment (Fig. 2d, upper panels). Similarly, for osteogenic cultures at 14th day of induction, Alizarin Red staining was performed and calcium depots were largely detected in induced samples while absent in un-induced controls (Fig. 2d, lower panels).

### Hyaluronic acid favors ASC embedding into lipoaspirate

After standardization of both lipoaspirate procedure and ASC isolation and before in vivo transplantation, we wanted to assess their combination using HA gel as carrier. It is known that regenerative medicine relies also on optimized interactions with cells and biomaterial as key factors for successful transplantation procedures [22, 23], in addition the use of autologous fat as a natural scaffold loaded with cells introduces an additional poorly explored bias possibly impacting cell and graft performance.

**Fig. 3:**
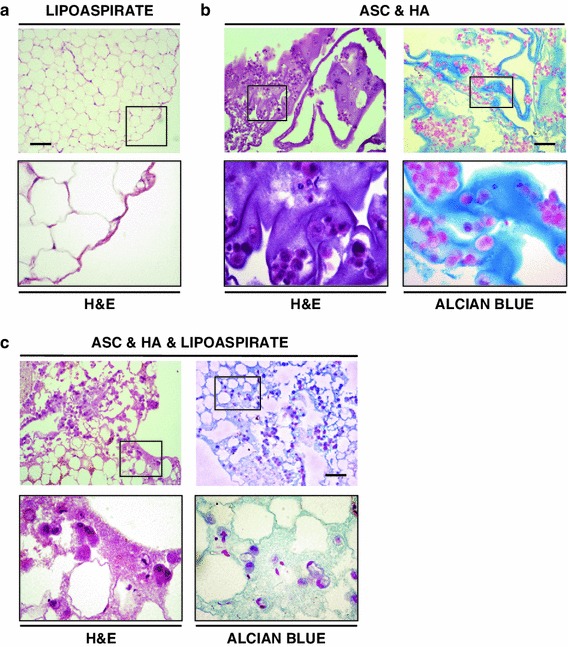
Histological features of the AFT-ASC composite. **a** Hematoxylin and Eosin (H&E) staining of lipoaspirate only specimens (*upper panel* and *inset*; *scale bar* 100 μm; *inset* ×4) revealing a comparable histological appearance of normal AT. **b** Representative photomicrographs of specimens consisting of ASC combined with hyaluronic acid (HA) and stained by H&E (*left panel* and *inset*) and Alcian Blue (*right panel* and *inset*). Intact ASC appeared organized as single elements or clusters within HA scaffold (*scale bar* 100 μm; *inset* ×4). **c** Representative H&E (*left panel* and *inset*) and Alcian Blue (*right panel* and *inset*; *scale bar*: 100 μm; *inset* ×4) stained specimens from a living bioscaffold composite consisting in AT combined with ASC and HA

Since lipoaspiration may cause AT disruption [5], we first verified the histological integrity of lipoaspirate alone after fixation and paraffin inclusion by microscopical examination and, as seen in Fig. 3a (upper panel and inset), lipoaspirate showed the histological appearance of a normal AT.

HA has been previously used as carrier for in vivo cell delivery in pre-clinical models for skeletal engineering while is poorly known for AT restoration [24, 25]. Therefore, we focused on the combination of ASC and HA to verify the impact of HA scaffold on ASC before the combination with lipoaspirate. Thus, we analysed the cell-gel combination by both H&E and Alcian Blue staining and, as seen in Fig. 3b (upper panels and insets), cells appeared as intact elements within the scaffold. ASC were organized either as single elements or as clusters inside of a dense amorphous substance positive for Alcian Blue as expected for a glycosaminoglycan containing matrix, such as HA. These findings demonstrated that cell-gel combination preserves cellular integrity and favors a rather homogenous distribution of cells. We then proceeded to combine ASC and HA with lipoaspirate to further verify the reciprocal distribution in order to proceed with the implantation. As seen in Fig. 3c, (upper panels and respective insets), lipoaspirate was combined with ASC plus HA. AT tissue was preserved, cells were maintaining their morphology and, in comparison with HA and ASC cells, clusters were here rarely detectable with more uniform distribution into lipoaspirate. In addition, Alcian Blue staining reveals how HA was embedding both the ASC and lipoaspirate suggesting an optimized distribution of cells and HA carrier in combination with fat graft prompting the use of AFT-ASC approach into in vivo studies.

### ASC preserve AFT integrity early after transplantation

Having combined carriers and ASC, we then performed transplantation procedures. AFT with or without autologous ASC were injected into right upper lips in selected animal groups (Fig. 1a–c). The procedures were well tolerated and no complications appeared after transplantation. Rabbits were healthy without changes in behavior and food intake. The early clinical monitoring at 7 days revealed an apparent increase in tissue consistency of transplanted side in both groups versus the un-transplanted internal matched control side.

**Fig. 4:**
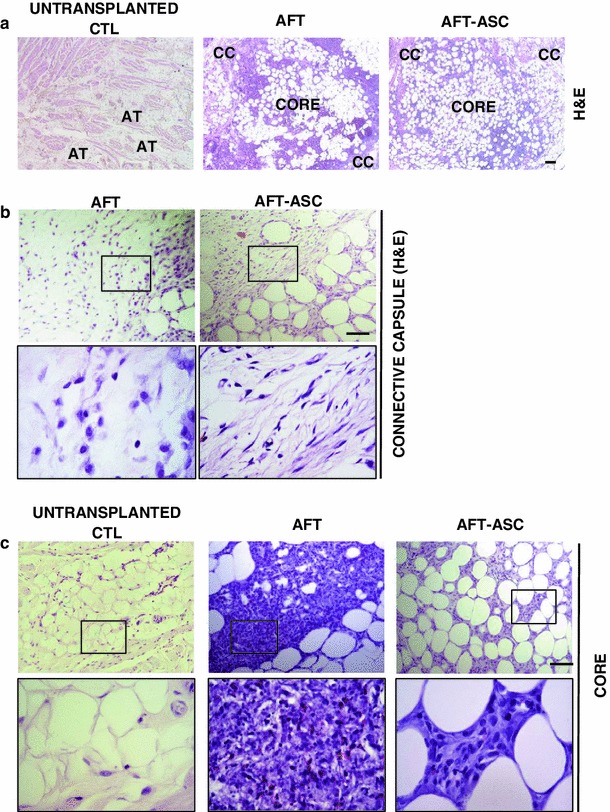
ASC preserve adipose tissue integrity early after transplantation. **a** Representative images of subcutaneous region in an untransplanted control (CTL, *left panel*), in AFT (*central panel*) and in AFT-ASC (*right panel*) transplanted specimens after 7 days from transplantation (H&E staining; *scale bar* 200 μm). While CTL samples were represented by connective tissue fibers, vessels, glands and nerve bundles along with adipose tissue (AT, *left panel*), in AFT and AFT-ASC groups, it was possible to identify an additional large area composed by a distinct inner core and outer connective capsule (CC) regions of graft material. **b** Connective capsule of AFT (*left panel* and *inset*) and AFT-ASC (*right panel* and *inset*) transplanted specimens showing similar histological appearance (*scale bar* 100 μm; *inset* ×4). **c** Representative photomicrographs of core grafts in AFT (*central panel* and *inset*) and AFT-ASC (*right panel* and *inset*) transplanted specimens. Inside AFT a dense and poorly organized extracellular structure including stromal elements, several cellular debris as well as numerous inflammatory cells was identifiable. On contrary, AFT-ASC core graft displayed more homogenously distributed adipocytes with a preserved morphology with more easily distinguishable stromal elements

After 7 days from transplantation, rabbits (*n* = 5/each) in both AFT and AFT-ASC groups were sacrificed and specimens were harvested. We first analyzed the un-transplanted internal control by H&E observing a sequential tissue stratification of cutis, subcutis, muscle tissue and mucosa (data not shown). As expected, rabbit subcutaneous tissue also revealed a loose meshwork of connective tissue fibers, large blood vessels, glands and nerve bundles (Fig. 4a, left panel). The spaces between extracellular networks were filled by adipocytes arranged in small lobules.

Focusing on both AFT and AFT-ASC transplanted specimens (Fig. 4a central and right panels), we identified an additional large area composed by distinct inner and outer regions. The external part was composed by a bundle of connective tissue (connective capsule) rich in vessels and lipidic cists. The central part of graft (core) was predominantly composed by adipocyte clusters into the extracellular matrix. In the attempt to compare AFT and AFT-ASC samples, we focused on the previously mentioned areas. The connective capsule of both groups (Fig. 4b) appeared similar, while the core graft of AFT specimens was characterized by a dense and poorly organized extracellular structure including stromal elements, several cellular debris as well as numerous inflammatory cells (Fig. 4c, central panel and inset). Furthermore, adipocytic elements were mostly represented by large lipid vacuoles next to far small irregularly shaped adipocytes. On contrary, AFT-ASC core graft displayed a more organized extracellular compartment with a substantial reduction of either debris or inflammatory elements (Fig. 4c, right panel and inset). Stromal cells were more easily distinguishable in a context between adipocytes that appeared more homogenously distributed with a preserved morphology without large abnormal elements. In addition, only in AFT-ASC group it has been possible to observe a vascularization. Collectively, this suggests that the inclusion of ASC into a grafted fat is associated with a better preservation fat tissue architecture at early time points from implantation.

### ASC lower necrotic areas inside AFT coupled with an improved angiogenesis

**Fig. 5:**
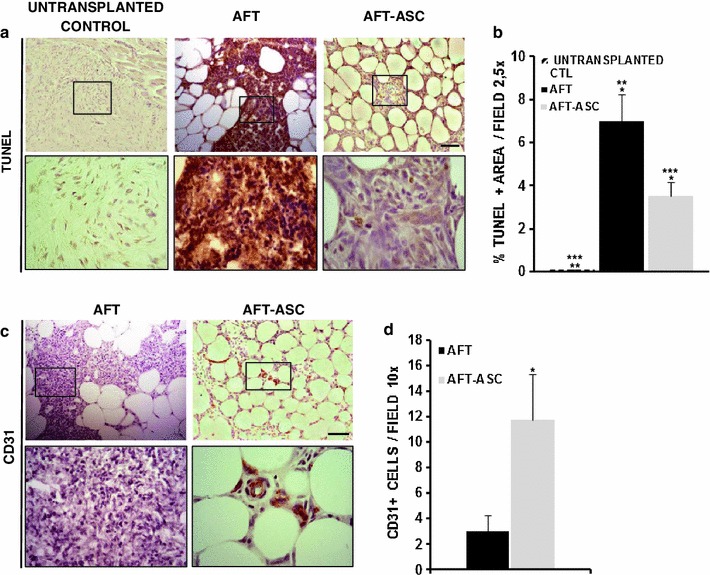
AFT-ASC composite reduces necrosis and increase angiogenesis early after transplantation. **a** Un-transplanted control (CTL; *left panel*), AFT (*central panel* and *inset*) and AFT-ASC (*right panel* and *inset*, *scale bar*: 100 μm; *inset* ×4) specimens stained by TUNEL assay. AFT transplants had prominent TUNEL-positive areas inside the core grafts in comparison with AFT-ASC transplants. **b** Quantitative measurement of TUNEL-positive areas in untransplanted CTL, and AFT-ASC specimens by ImageJ analyses. The TUNEL+ areas for AFT-ASC samples were statistically reduced in comparison of AFT samples. *p = 0.013; **p = 0.2 × 10^−3^; ***p = 0.4 × 10^−3^. **c** Anti-CD31 immunohistochemical staining of AFT (*left panel* and *inset*) and AFT-ASC (*right panel* and *inset*, *scale bar* 100 μm; *inset* ×4) specimens. AFT-ASC core graft had a greater angiogenesis versus AFT core graft. **d** Quantitative measurement of CD31-positive vessels for both FAT and AFT-ASC specimens. AFT-ASC core graft displayed a statistically increase in small vessels in comparison of AFT core graft. *p = 0.025

In the attempt, to clarify the reported findings, we then focused on two main parameters: apoptosis and vascularization. At 7 days post-transplantation, by ImageJ software analysis, we quantify the presence of apoptotic and necrotic areas inside graft cores by TUNEL. Similarly to the histological features observed during fat necrosis [26], AFT transplants had a more prominent TUNEL+ areas (Fig. 5a, central and inset panel; Suppl. Fig. 1 middle and inset) inside the core grafts in comparison with AFT-ASC group (Fig. 5a, right and inset panel; Suppl. Fig. 1 lower and inset panel), In particular, ASC/HA co-transplantation with AFT was followed by a 50 % reduction in TUNEL+ areas of grafted tissue versus AFT alone (3.51 ± 0.64 vs. 6.38 ± 1.23 %; *p* = 0.01; Fig. 5b).

These data demonstrated a better preservation of fat graft viability by the addition of ASC, with a protective effect early after implantation.

Since vascularization is known to protect against ischemia related-apoptosis, we then sought to evaluate the presence of vessels inside the graft areas in both groups assessing CD31+ endothelial cells. Seven days after transplants, in the graft cores of AFT-ASC (Fig. 5c, right panels), the quantity of CD31+ cells was 11.7 ± 3.5/100× high power field versus 3 ± 1.26 obtained after scoring of AFT specimens (Fig. 5c, left panels; *p* = 0.02). We also considered the CD31 levels in the connective capsule of both groups with evidence of a higher CD31+ cells versus the graft core and, while AFT-ASC had a better performance than AFT (23.19 ± 4.38 and 17.57 ± 5.31 respectively), the difference was not significant (*p* = 0.40).

Collectively, these findings proved the capacity of HA combined with ASC loaded into AFT to be associated by new high vascularization inside the graft core early after transplantation, suggesting a better vasculogenesis that may also explain a reduced necrosis into AFT-ASC.

### ASC improve AFT performance 3 months post-transplantation

**Fig. 6:**
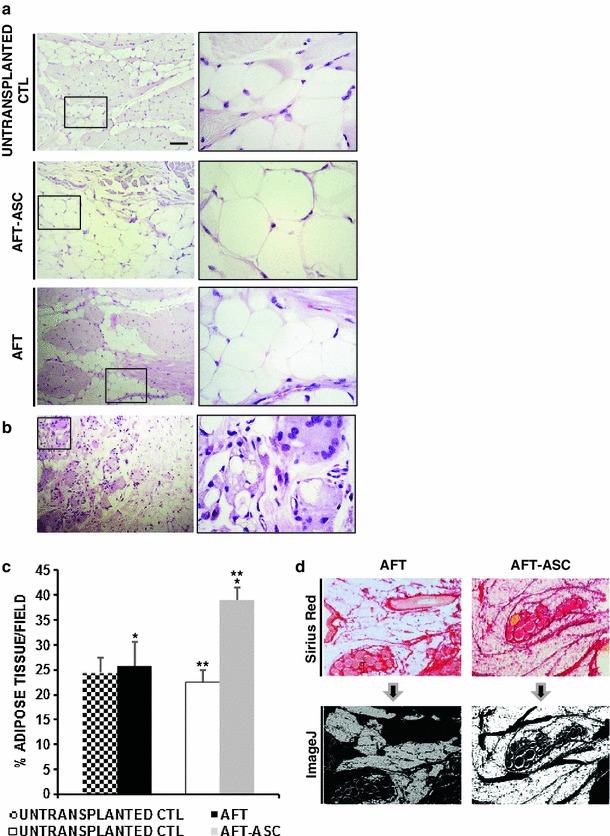
AFT-ASC composite is associated with a significant lipopreservation 3 months after transplant. **a** Subcutaneous regions of untransplanted CTL (*upper panel* and *inset*), AFT (*middle panel* and *inset*) and AFT-ASC (*lower panel* and *inset*, *scale bar* 100 μm; *inset* ×4) specimens after 3 months post transplantation. **b** AFT specimen stained by H&E and characterized by the presence of discrete areas with syncytial-like granulomatous structures associated with inflammatory cells (*scale bar* 100 μm). **c** Quantitative measurement of preserved adipose tissue in both AFT and AFT-ASC specimens 3 months after transplantation and respective untransplanted CTL after Sirius Red staining and ImageJ analyses. *p = 0.021; **p = 0.41 × 10^−4^. **d** Representative images of Sirius Red staining (*upper panels*) and their elaboration (*lower panels* by ImageJ) in both AFT and AFT-ASC specimens

To evaluate in a longer observation time the performance and safety of AFT assisted by ASC, right upper lips, as transplanted sample, and left upper lips, as un-transplanted control were processed for histology 3 months after transplantation. All specimens displayed a more prominent subcutaneous tissue in comparison with the 7 days time point, most probably due to a consequent increase in body fat with age (not shown), and suggesting a regular growth of treated animals. As expected, subcutaneous region of un-transplanted control was composed by normal-shape adipocytes grouped into larger lobules (Fig. 6a, upper panels). This finding was also detectable in both grafted groups, with or without ASC, and the anatomical compartmentation with a capsule and a core graft observed at 7 days was not anymore detectable (Fig. 6a, middle and lower panels). Necrotic areas and lipidic cysts, clearly present in group AFT at 7 days, were negligible and appeared replaced by sporadic presence of a syncytial-like granulomatous structures associated with inflammatory cells (Fig. 6b). This finding was not observed in AFT-ASC group and may be represent a focal points of tissue reabsorption as a consequence of events observed early after transplantation, as previously described for lipophagic necrosis [26]. Due to a possible remote chance of primary wild type progenitor cell transformation after transplant, AFT-ASC were further considered in the attempt to identify proliferative or dysplastic features. No evidence of abnormalities were detected in ASC enriched samples, supporting the safety of our approach at 3 months from the transplant.

Having observed the principal AT features after 3 months, we wanted to verify if the AFT-ASC obtained specimens had more preserved adipose volume then the controls. Sirius Red assay was then implemented comparing the average AT area (Fig. 6c, d). The untransplanted matched controls (left lips) for AFT and AFT-ASC groups displayed a similar amount of Sirius red negative AT (24.46 ± 3.07 vs. 22.54 ± 2.35 respectively; *p* = 0.6). Comparing AFT transplanted samples and their matched untransplanted controls, similar results were obtained (25.68 ± 4.97 vs. 24.46 ± 3.07 respectively; *p* = 0.83) suggesting a 95 % resorption of transplanted AT whose level returned to a pre-transplant time, as reported [3, 4]. On contrary, there was a significant difference in amount of AT between AFT-ASC transplanted samples and their un-transplanted controls (38.92 ± 2.63 vs. 22.54 ± 2.35 respectively; *p* = 4.14 × 10^−5^). This was also confirmed against AFT group where a significant lower amount of AT was detected (38.92 ± 2.63 and 25.68 ± 4.97 respectively; *p* = 0.02), with a relevant better performance in AFT-ASC transplanted sites versus the AFT only.

## Discussion

ASC are promising regenerative tools based on either their intrinsic differentiation capacities or on the secretion of trophic soluble factors [27]. For these reasons they have been introduced into various regenerative medicine applications [14]. We here report the impact of purified autologous ASC in the amelioration of AFT performance.

Clinical indications for AFT have been moving from soft tissue restoration for aesthetic purposes to the treatment of more challenging congenital and acquired pathological conditions associated with severe morbidity such as tissue loss after trauma, burns, post-tumor resections, HIV-related lipodystrophies and for rare types of congenital diseases, where the lack of fat may be often associated with deathly metabolic syndromes [28]. Since the pioneeristic introduction of AFT into clinical practice by Neuber, techniques to improve the procedure have been implemented [1, 29]. However, grafts take remains highly variable and volumes significantly decrease over time making a long-term outcome sub-efficient and difficult to predict [30].

While the reasons for this poor AFT outcome are still under investigation, AFT approaches currently require additional tissue implantations aiming to optimization [29]. This implies the need of more available autologous AT, the increase of surgical risks with additional negative impacts on procedures. Moreover, the lack of available AT may represent a major limitation for repeated procedures, in particular during cachexia in cancer patents and lipodystrophies.

These limitations led to the development of an approach based on freshly isolated SVF enriching the AFT [8]. Preclinical studies reported that SVF addition improves long-term viability and quality of retained fat [8, 31, 32]. Nevertheless, the internal reabsorption rate for SVF enriched fat has been highly variable with a reduction up to fourfold from the starting volumes [31, 32]. This variable outcome may be linked to the distinct procedures and tools used to purify SVF. Additionally, since SVF composition is heterogeneous [12] and composed by endothelial, blood and progenitor stromal cells including variable amount of ASC, their different quantities may also impact the therapeutic outcome of SVF assisted AFT. Based on these findings, we sought to use a purified population of adipose progenitors to enrich the total amount of regenerative cells within the fat transplant by an ASC assisted AFT. For the first time to our knowledge, defined numbers of ASC combined with AT and HA were introduced into an autologous animal model to challenge the therapeutic potential of ASC and begin to understand the biological bases of fat graft performance after transplantation.

The combined transplantation approach into New Zealand rabbits was selected because of their small animal size and presence of defined areas of subcutaneous AT allowing easy access by either exeresis or liposuction [15, 33]. One of the major limitations studying ASC in small animal models may be the quantity of AT starting volume to isolate and expand the desired cell yield, in particular when multiple harvests are required. While this is not generally an issue in humans, the limited body fat obtainable in rabbits may impact the quantity of ASC. Therefore, enzymatic digestion procedures and amplification were here optimized to generate sufficient numbers of progenitors starting from 150 mg of AT in comparison with previous procedures that rely on at least 10 g of AT [33, 34]. Beside an improved isolation step, by ex vivo amplification we were able to obtain an average of 10^8^ ASC for each ml of lipoaspirate in 20 days, while there have been previously described expansions between 0.2–4 × 10^6^/ml after 40 days of culture [33, 35, 36]. These differences may be related to distinct factors, such as the anatomic site of harvested AT and the in vitro expansion conditions. It is well known that different sampling sites could generate various amount of ASC in humans and this may be similar in rabbit [37]. In this study, AT was obtained from subcutaneous inguinal fat, indirectly confirming very recent data on a better ASC performance from rabbit inguinal fat pad [38]. Dealing with culture conditions and as reported for human marrow MSC [39], the introduction of a FBS free protocol had a positive impact on ex vivo amplifications, as also showed by the differentiation assays where purified rabbit ASC showed multipotential capacities.

To further validate the mesenchymal phenotype of obtained cells, several antibodies were applied, also based on a limited anti-rabbit availability. The CD29, CD90 and, surprisingly, CD49e were expressed by rabbit ASC while others, generally used for human MSC designation such as CD73, CD105 showed a low or negligible expression, partly confirming what has been previously described [40] and additionally indicating that rabbit ASC may have a distinct phenotypic profile versus human ASC that requires further studies. In particular, CD90 expression seems much lower in rabbit versus human cells. To confirm these data, our CD90 expression was max 41.3 % with a mean value of 32.5 %. More expressed and closer to human ASC appeared the CD29 levels that were capable to reach approximately. 90 % in line with other literature data [41, 42].

Having selected the animal model, the fat harvest procedure and obtained a purified population of ASC, we focused on cell delivery into fat graft. It has been previously shown that lipoaspirate water content may interfere with cells and AT interactions, inducing sub-optimal cellular integration into transplanted fat with loss of cellular component from transplanted site, additionally resulting in unresolved complication, such as ectopic fibrosis, cysts and calcifications [9, 43]. To abrogate these risks and optimize transplantation procedure, we wanted to introduce a strategy that combines cells, AT and a carrier to reduce ectopic cellular migration and post-surgical complications. Thus, we focused on hyaluronic acid (HA) as possible gel carrier for ASC since it also reduces the water content in the graft for its polyanionic hygroscopic nature [44]. HA hydrogels alone have been extensively introduced as injectable scaffolds for soft tissue and cartilage repair since they do not retain organ specificities and cause immunological reactions. Moreover, HA-based hydrogels have been used for cell delivery in several pre-clinical models, such as osteochondral defect repair, vocal folds restoration and stroke [24]. Recently, Alghoul et al. [45] hypothesized that HA may itself improve graft performance, and introducing relevant amount of HA into transplanted fat (up to 50 % in the total volume) they observed transient impact on graft survival. In the current study, we cannot exclude that the HA alone contained in our graft may have a protective effect, nevertheless our limited content of HA (5 %) was considered negligible to impact alone on graft survival. In addition, the exclusive use of relevant amount of HA will be unlikely transferred into clinic suggesting the need of other active elements, such as ASC.

HA as gel scaffold has been also used to deliver ASC for fat engineering in a pig model aimed to further support AT, although revealing a transient effect [25]. Mixing HA and rabbit ASC, we found that HA is not detrimental for cell survival and proliferation in vitro (data not shown), this was particularly true when HA was additioned with autologous rabbit serum before implantation allowing the composite HA-ASC-AT.

The concept of a tissue-engineering injectable composite was proposed by Burg and Boland and applied for cartilage engineering and fat reconstruction [24]. In these studies, injectable synthetic composites were loaded with HA combined with pre-adipocytes for soft tissue regeneration. Our strategy moves from this concept and wants to introduce a living composite as a more natural delivery vehicle for stem cells, additionally providing a more physiological microenvironment after transplantation possibly facilitating cell survival and stable graft take. The major limitations of this approach may be represented by the exclusive autologous use and by the need of slightly more complex surgical procedures that require a first step for ASC isolation and a second approach for cell purification and bioscaffols implantation. However, a standardization of ASC isolation from minimal quantity of AT may limits patient discomfort, avoiding demanding surgical approaches.

The in vivo studies allowed us to begin to understand AFT performance in a time course manner after transplantation. We first investigate the presence of fat necrosis within the AFT and AFT-ASC grafts at 7 days after transplantation. Necrosis most probably represents the pivotal event that reduces AFT performance so that the overall incidence of fat necrosis may involve more than 1/3 of all breast reconstruction by AFT [7, 26, 46]. In addition, AT necrosis can mime breast cancer recurrence both clinically and radiologically, thus the major challenge to improve AFT performance seems the reduction of necrosis.

Several authors investigated different pharmaceutical approaches to increase fat graft survival, with variable outcomes [45, 47, 48]. In our study, the introduction of a AFT-ASC approach with ASC significantly reduce early necrosis associated with a better preservation of histological features within grafted AT. The role of ASC in having a protective factor against apoptosis has been under investigation in different fields. In particular, the production of soluble mediators such as growth factors, neurotrophic and pro-angiogenic factors have been described as keys [49, 50].

Among those, we focused on angiogenesis observing significantly higher number of CD31+ endothelial cells in AFT-ASC grafts versus AFT. It has been reported that fat graft survival is mainly dependent on successful vascularization so that adipogenesis is associated with capillary angiogenesis allowing adipocyte differentiation within clusters of endothelial and stromal cells [4]. While some data suggest that adipose progenitors enriching AFT may differentiate into PECAM-1+ endothelial cells within the graft [32], others indicate a promotion of angiogenesis by release of factors such as VEGF, excluding a direct participation in vasculogenesis [8, 31]. In the current study we were limited in dissecting the pivotal mechanisms behind a greater endothelial presence in AFT-ASC due to the autologous model, therefore gene marking will be required to clarify whether the impact of transplanted cells into the vascular graft compartment may be due to differentiation, release of soluble factors or both.

The prolonged in vivo study allowed us to additionally verify the status of the graft at 3 months post-transplantation observing a robust retainment in AT volume for AFT-ASC grafts compared with both untransplanted control and AFT that shown a 95 % AT reabsorption returning to base-line levels similarly to the untransplanted control. At 3 months AFT grafts additionally displayed granulomatous structures and fibrotic areas surrounded by inflammatory elements as described for the concomitant presence of fat necrosis and granulation tissue with inflammation [7, 46]. This leads to suppose that granulomatous tissue observed at 3 months in AFT grafts could represent focal area of pre-existing fat reabsorption due to the intensive necrosis observed 7 days after transplantation, as described for lipophagic necrosis in later stages of ischemic necrosis [26]. These findings suggest that the early protective effect of ASC in reducing necrosis may generate a long term impact in AFT performance allowing a better preservation of AT and avoiding histopathological feature that may also generate detrimental imaging interpretations. On the same line and dealing with safety, ASC included into AFT were never associated by histological abnormalities suggestive for transformation, such as evidence of macroscopic irregular AT growth or clusters of monomorphic cellular elements inside fat graft without any signs of animal mortality and morbidity.

In conclusion, introducing a novel AFT-ASC approach we here further optimized sub-cutaneous AT regeneration, reducing early negative events impacting AFT with a significant better graft performance. These data confirm the potential of purified adipose progenitors in more stably supporting regenerative medicine approaches for severe pathological conditions affecting subcutaneous soft-tissues.

## Electronic supplementary material

Below is the link to the electronic supplementary material.

Supplementary material 1 (DOCX 1510 kb)

Supplementary material 2 (DOCX 1391 kb)
