# Supplementary material for: Adipose stromal/stem cells assist fat transplantation reducing necrosis and increasing graft performance
Source: Apoptosis. 2013 Jul 5;18(10):1274–89. doi: 10.1007/s10495-013-0878-7 (PMC3775159; doi:10.1007/s10495-013-0878-7)
Supplement: Supplementary file 1 — Supplementary material 1 (DOCX 1510 kb) [file 10495_2013_878_MOESM1_ESM.docx]

**Piccinno et al. Supplementary Figure 1**

**
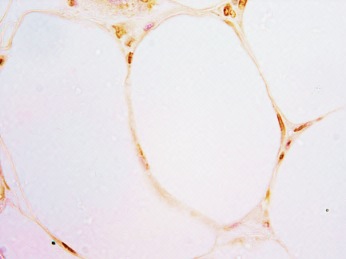

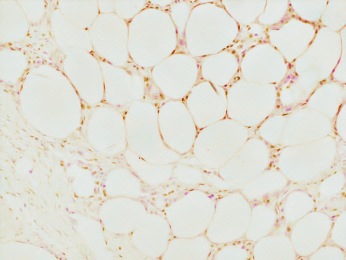
**

**TUNEL**

**POSITIVE CONTROL**


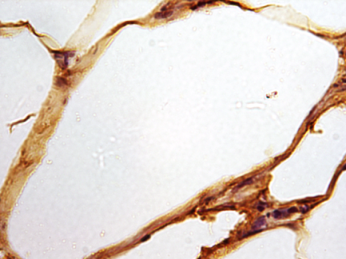


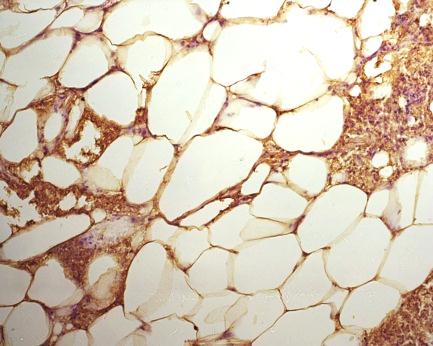


**AFT**


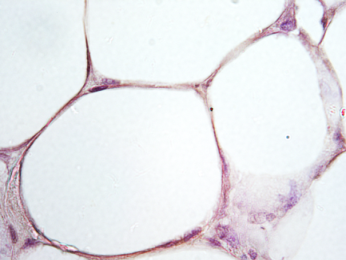

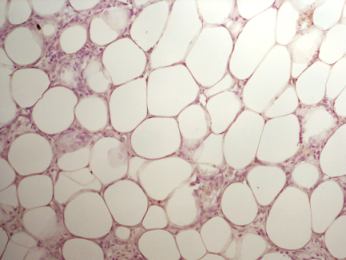


**AFT-ASC**

**Supplementary Figure 1. Tunel assay discriminates dead and viable adipocytes.** Positive control (upper panel and inset), AFT (middle panel and inset) and AFT-ASC (lower panel and inset, scale bar: 100µm; inset 4x) specimens stained by TUNEL assay. AFT-ASC core graft displayed viable adipocytes with nuclei that were negative for staining. On contrary, AFT core graft showed dead adipocytes with TUNEL positive signals.
