# Supplementary material for: Adipose stromal/stem cells assist fat transplantation reducing necrosis and increasing graft performance
Source: Apoptosis. 2013 Jul 5;18(10):1274–89. doi: 10.1007/s10495-013-0878-7 (PMC3775159; doi:10.1007/s10495-013-0878-7)
Supplement: Supplementary file 2 — Supplementary material 2 (DOCX 1391 kb) [file 10495_2013_878_MOESM2_ESM.docx]

**Piccinno et al. Supplementary Figure 2**


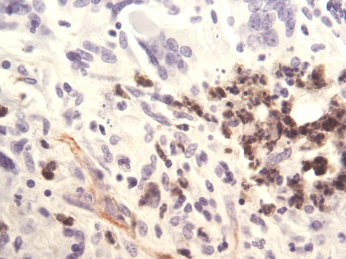


**anti-Mac(black) & anti-CD31(red)**


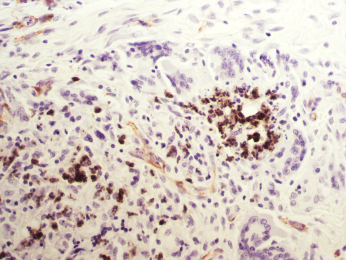


**20x**

**40x**

**The adopted anti-CD31 staining is selective for vascular endothelial cells in rabbit**. To exclude that the observed and scored CD31+ cells were macrophages, a double immunohistochemistry staining with both CD31 (in red by NOVARED staining) and anti-macrophage antibody (MAC387, Abcam; 1:750 in black by Nickel-DAB staining) was performed. Endothelial cells could be easily detected as red only colored vessels structures (arrowheads) while macrophages are identified as dark elements alone or in clusters in the specimens (arrows). Two different magnifications are here reported.
